# The distinct and overlapping phenotypic spectra of *FOXP1* and *FOXP2* in cognitive disorders

**DOI:** 10.1007/s00439-012-1193-z

**Published:** 2012-06-27

**Authors:** Claire Bacon, Gudrun A. Rappold

**Affiliations:** Department of Human Molecular Genetics, University of Heidelberg, Im Neuenheimer Feld 366, 69120 Heidelberg, Germany

## Abstract

Rare disruptions of *FOXP2* have been strongly implicated in deficits in language development. Research over the past decade has suggested a role in the formation of underlying neural circuits required for speech. Until recently no evidence existed to suggest that the closely related *FOXP1* gene played a role in neurodevelopmental processes. However, in the last few years, novel rare disruptions in *FOXP1* have been reported in multiple cases of cognitive dysfunction, including intellectual disability and autism spectrum disorder, together with language impairment. As FOXP1 and FOXP2 form heterodimers for transcriptional regulation, one may assume that they co-operate in common neurodevelopmental pathways through the co-regulation of common targets. Here we compare the phenotypic consequences of *FOXP1* and *FOXP2* impairment, drawing on well-known studies from the past as well as recent exciting findings and consider what these tell us regarding the functions of these two genes in neural development.

**Electronic supplementary material:**

The online version of this article (doi:10.1007/s00439-012-1193-z) contains supplementary material, which is available to authorized users.

## Introduction

Forkhead box (FOX) proteins are an evolutionarily ancient family of transcription factors characterised by a highly conserved forkhead DNA-binding domain. Despite the similarity in the DNA-binding domain, FOX proteins have a wide range of important biological functions (Hannenhalli and Kaestner 2009). Most FOX proteins bind to their target DNA sequences as monomers, except members of the FOXP subfamily, which includes FOXP1–4. FOXP proteins are somewhat atypical as they also possess a zinc finger and leucine zipper domain, both of which mediate interactions with other proteins, thus allowing FOXP proteins to form homo- and heterodimers to facilitate their binding to DNA for transcriptional regulation (Li et al. 2004). In the past, members of the FOXP subfamily have been implicated in various human diseases (Bennett and Ochs 2001; Jin et al. 2010; Lai et al. 2001), but only *FOXP1* and *FOXP2* have been linked to cognitive disorders so far.

Perhaps the most prominent of the *FOXP* genes is *FOXP2* (OMIM 605317), whose involvement in speech and language acquisition has now been studied for more than 10 years (Fisher and Scharff 2009). *FOXP2* and language development has generated extreme interest as the acquisition of spoken language is central to what makes us human. In the last 2 years, evidence has emerged that implicates *FOXP1* (OMIM 605515), the closest relative of *FOXP2* (64 % total protein sequence identity, 89 % in the forkhead domain), in the pathology of human cognitive disorders, which involve language impairment. FOXP1 and FOXP2 are already known to co-operate in the regulation of non-neural developmental processes (Shu et al. 2007) and it will be interesting to validate whether this co-operation extends to brain development. A comparison of the clinical phenotypes caused by *FOXP1* and *FOXP2* disruption would provide important steps towards uncovering those regions of neuropathology in which these genes play a role.

## *FOXP2* mutations are implicated in developmental verbal dyspraxia

Foxp2 contributes to lung, heart and oesophagus development (Shu et al. 2001, 2007), but the most well-known role of this gene is in the development of speech and language (Fisher and Scharff 2009; Newbury and Monaco 2010). Human *FOXP2* was first linked to language development in 2001, when a heterozygous missense mutation in the forkhead domain (R553H) was found to be causative for an inherited language disorder in a large 3-generation pedigree known as the KE family (Lai et al. 2001). The KE family has been extensively studied and the phenotype is now well defined, affecting expressive, receptive and written language (Table 1). A core feature of the disorder is a difficulty in the learning and production of co-ordinated sequences of orofacial movements, which impairs the production of fluent speech (Vargha-Khadem et al. 1995; Watkins et al. 2002a). Therefore the affected members have a primary diagnosis of developmental verbal dyspraxia (DVD), which is also known as childhood apraxia of speech (CAS). Studies on the KE family have also included formal evaluations of non-verbal intelligence and the average IQ of affected KE family members is lower than that of unaffected individuals (Fisher et al. 1998; Vargha-Khadem et al. 1995; Watkins et al. 2002a), raising the possibility that the missense mutation in the *FOXP2* gene also impacts other cognitive functions. Nevertheless, the most prominent and consistent features of the disorder are in the domain of speech and language, with more severe and wide-ranging effects on verbal skills than on non-verbal cognition (Watkins et al. 2002a).

**Table 1:** Summary of clinical phenotypes of patients reported with heterozygous intragenic mutations, deletions and disruptions of *FOXP2* (in chronological order)

References	*FOXP2* variant	IQ	Clinical information
Vargha-Khadem et al. (1995, 1998)^a^	R553H (KE)	86 affected, 104 unaffected	Impaired speech production. Grammar defects. Impaired orofacial praxis. Articulatory impairment. Abnormal activation in motor-related areas during word repetition (PET). Caudate nucleus is structurally abnormal and smaller in affected members (MRI)
Fisher et al. (1998)^a^	R553H (KE)	63–101 affected, 82–118 unaffected	NR
Watkins et al. (2002a, b)^a^	R553H (KE)	83; affected (2), 98; unaffected (3)	Expressive, receptive and written language impaired. Impaired articulation is core deficit Affected members had reduced volume and significantly less grey matter bilaterally in the caudate nucleus (voxel-based morphometry of MRI)
Liegeois et al. (2003) ^a^	R553H (KE)	NR	Abnormal activation of Broca’s area and putamen (fMRI)
MacDermot et al. (2005) ^a^	R328X	NR	DVD. Repetitive and expressive language scores 3 SD below mean. Problems with articulation. Affected sibling also carried variant
	Q17L	NR	DVD. Affected sibling did not carry variant
	Insertion leading to an expansion of the polyglutamine tract	NR	DVD. Affected sibling did not carry variant
Lai et al. (2000) ^b^	t(5;7) (q22;q31.2) balanced translocation	NR	Language impairment. DVD. Mild delay in mental development at 3.5 years
Shriberg et al. (2006) Tomblin et al. (2009)^b^	t(7;13) (q31.1;q13.2) balanced translocation	95 and 87	Both mother and daughter have spastic dysarthia and DVD. Expressive and receptive language and grammatical ability impaired
Feuk et al. 2006)^b^	t (3;7) (q23;q31.2) translocation	NR	DVD
Feuk et al. (2006)^c^	Five 7q31 dels: 3 × 15 Mb, 13 Mb and 11 Mb	3 patients below average	All five patients had DVD as well as speech delay and an articulation disorder. Two patients had general developmental delay. One patient was reported to have ASD, another to be ‘ASD-like’
Zeesman et al. (2006)^c^	Del 7q31.2–q32.2		Female patient. DVD. Cognitive abilities ranged from below average to average. Did not meet criteria for autism
Lennon et al. (2007)^c^	9.1 Mb del 7q31.2–7q31.31		Female patient. DVD. Moderate intellectual disability. Did not meet criteria for autism
Zilina et al. (2011)^c^	~8.3 Mb del 7q31.1–q31.31., inherited from mother	88	Female patient. Moderate developmental delay. Poor vocabulary. Orofacial motor defects. Some autistic features. Affected mother has speech delay and DVD
	6.5 Mb del 7q31.1–q31.2., inherited from mother	NR	Developmental delay in all areas. Mild ataxia. Pronunciation difficulties and poor vocabulary. Affected mother has intellectual disability, DVD and mood disorder
Rice et al. (2011)^c^	1.57 Mb del (*FOXP2*, *MDFIC*, *PPP1R3A*). Inherited from mother	75 patient, 89 mother	Male patient. DVD. Mother’s early verbal development similar to the patient’s
Palka et al. (2012)^c^	14.8 Mb mosaic del	71	Female patient. DVD. Developmental delay. Normal brain MRI. Mild psychomotor retardation. Severe language delay. Impaired receptive, expressive, comprehensive and written language

All IQ scores are non-verbal

*DVD* developmental verbal dyspraxia, *ASD* autism spectrum disorder, *NR* not reported

^a^Rows represent intragenic mutations leading to amino acid changes and insertions

^b^Rows represent translocations

^c^Rows represent deletions

The presence of just one de novo mutation within the coding region of *FOXP2* did not, by itself, provide compelling evidence that *FOXP2* contributes to the pathology of DVD. To assess the contribution of heterozygous *FOXP2* mutations in patient samples independent from the KE family, *FOXP2* was screened in 49 unrelated children diagnosed with DVD (MacDermot et al. 2005). Three different variants altering the FOXP2 protein sequence in three individual patients were identified. The most interesting of these variants was a heterozygous nonsense mutation (not found in 252 controls) which yielded a stop codon at position 328 of the FOXP2 protein, resulting in either a truncated protein missing the functionally important forkhead, leucine zipper and zinc finger domains or a complete loss of FOXP2 due to nonsense mediated RNA decay. The child had a diagnosis of DVD and displayed articulation problems along with impaired receptive and expressive language development. The same mutation was also detected in an affected sibling as well as the mother, who was reported to have a history of speech difficulties. This finding offered further evidence that *FOXP2* is of pathological importance in DVD in the absence of other cognitive disorders, which were exclusion criteria in this study.

Various large-scale 7q31 deletions that include the *FOXP2* gene, ranging from 1.57 Mb to 15 Mb in size, have also been reported in patients with DVD (Feuk et al. 2006; Lennon et al. 2007; Palka et al. 2012; Rice et al. 2011; Zeesman et al. 2006; Zilina et al. 2011). DVD was reported in every case and most of the reports concluded that impaired language was due to the loss of *FOXP2*. However, the large size of these deletions encompassing several to many genes is a complication for understanding the links between phenotypes and *FOXP2* dysfunction. Translocation breakpoints directly disrupting the *FOXP2* locus in patients diagnosed with DVD provided more clear-cut support for the relevance of this gene to speech and language pathology (Table 1) (Feuk et al. 2006; Lai et al. 2000, 2001; Shriberg et al. 2006; Tomblin et al. 2009).

The reported *FOXP2* variants are rare but have not been found in normal individuals from the 1,000 genome project and taken together, three translocation breakpoints disrupting *FOXP2* (Feuk et al. 2006; Lai et al. 2000; Shriberg et al. 2006), one missense mutation (Lai et al. 2001) and one nonsense mutation (MacDermot et al. 2005) independently associated with a DVD phenotype provide strong genetic evidence for the role of *FOXP2* in the clinical phenotype (summarised in Table 1).

## Evidence for a role of *FOXP1* in neurodevelopmental disorders

Foxp1 has been associated with a wide range of functions including development of the lung, heart, oesophagus, immune system and spinal motor neurons, as well as cancer (Banham et al. 2001; Dasen et al. 2008; Hu et al. 2006; Jepsen et al. 2008; Palmesino et al. 2010; Rousso et al. 2008; Shi et al. 2004; Shu et al. 2001, 2007; Wang et al. 2004). It has been known for some time that Foxp1 is expressed in particular neuronal subpopulations in the developing brain (Ferland et al. 2003), but its precise roles in brain development have not been defined. Given that the expression of *Foxp1* and *Foxp2* overlaps in certain areas of the developing brain (Ferland et al. 2003; Teramitsu et al. 2004) and that Foxp1 and Foxp2 are able to form heterodimers for transcriptional repression (Li et al. 2004), it is reasonable to hypothesize that *FOXP1* could also be involved in the pathology of DVD. However, in a screen of the entire coding region of *FOXP1* in 49 DVD patients, (the identical cohort used in the *FOXP2* screening described earlier), no potentially disease-causing variants were found (Vernes et al. 2009). Although the patient cohort was small, it suggested that, unlike *FOXP2*, mutations in *FOXP1* are not sufficient to cause DVD in isolation. Instead, various screening studies carried out within the last few years have revealed that *FOXP1* may be of more global importance in a range of neurodevelopmental disorders, which includes but is not restricted to speech and language disorders.

The first hint that *FOXP1* may be involved in neurodevelopmental disorders was provided by a heterozygous deletion in 3p14.1, which affected *FOXP1*, *EIF4E3*, *PROK2* and *GPR27*, in a patient with speech delay, hypertonia and additional phenotypes (for details see Table 2) (Pariani et al. 2009). The authors optimistically attributed many of the patient’s symptoms including the speech delay to the disruption of *FOXP1*, but the contribution of *PROK2* and *GPR27* to the phenotype cannot be ruled out, especially considering that both genes were previously implicated in developmental retardation (Petek et al. 2003). More convincing evidence was provided when a deletion exclusively affecting the *FOXP1* gene was found in a male child with impaired language acquisition and motor development delay (Carr et al. 2010). Unfortunately, a confounding factor in this study was the presence of a Chiari I malformation (cerebellar tonsil abnormality) in the patient, which may have contributed to the delay in motor and speech development, therefore convincing evidence for a role of *FOXP1* in cognitive disorders was still lacking.

**Table 2:** Summary of clinical phenotypes of patients reported with heterozygous disruptions in *FOXP1* (in chronological order)

Reference	*FOXP1* variant	IQ	Clinical information
Pariani et al. (2009)^a^	785 kb del (*FOXP1, EIF4E3*, *PROK2* and *GPR27*)	NR	Male patient. Gross motor delay. Contractures. Blephharophimosis Hypertonia. Speech delay; vowel sounds but no words at 23 months
Carr et al. (2010)^a^	~1.0 Mb del	NR	Male patient. Gross motor delay. Chiari I malformation, dysmorphic but intact corpus callosum and mild hypoplasia of the cerebellar vermis (MRI). Speech delay, difficulty producing multisyllabic speech, limited verbal output. Effective use of sign language to compensate problems with verbal expression. No deficits in oromotor co-ordination
Horn et al. (2010)^a^	498 kb del (all but first coding exon)	<50	Male patient. Gross motor delay. Speech delay; first words at 3.5 years, combined words at 7 years. Expressive language more affected than receptive language. Articulation problems and poor grammar. No brain abnormalities (MRI, EEG)
	659 kb del (entire coding region)	<50	Female patient. Gross motor delay. Speech delay; first vocalising at 4 months, first words at 3.5 years, combined words at 5 years. Expressive language more affected than receptive language. Articulation problems and poor grammar. No brain abnormalities (MRI, EEG)
	1,047 kb del (entire coding region)	50	Male patient. Gross motor delay. Speech delay – first vocalising at 12 months, first words at 3.5 years, combined words at 5.5 years. Expressive language more affected than receptive language. Articulation problems and poor grammar. No brain abnormalities (MRI, EEG)
Hamdan et al. (2010)	~390 kb del (exons 4–14 of *FOXP1a*)^a^	58	Female patient. Global developmental delay. Severe language impairment, expressive language of 4.5 years at 15 years of age. Receptive language more developed. No deficits in oromotor co-ordination reported. Autistic features but below threshold for autism diagnosis. Aberrant behaviours with social withdrawal, anxiety, aggression, irritability
	R525X^b^	48	Male patient. Global developmental delay. ASD. Severe language impairment; expressive language of 1 year 11 months at 9 years of age. Performed at age 3 years 7 months in auditory comprehension tasks. No defecits in oromotor co-ordination reported. Aberrant behaviours with irritability, hyperactivity, stereotypy, obsessions and compulsions, self-injurious behaviour
O’Roak et al. (2011)^b^	p.Ala339Ser*fs*X4	34	Male patient. Language (phrases) delay. ASD. Aberrant behaviours include lethargy, hyperactivity, inappropriate speech
Talkowski et al. (2012)^c^	t(3;10) (p13;q21.2) balanced translocation	NR	Global developmental delay. Speech delay. Bilateral inguinal hernia. Spina bifida oculta. Dysmorphic features

All IQ measurements are non-verba

*NR* not reported, *ASD* autism spectrum disorder

^a^Rows represent deletions

^b^Rows represent intragenic mutations

^c^Row represents a translocation

Shortly afterwards, a large-scale screen for copy number variations (CNVs) in 1,523 patients with intellectual disability, uncovered three de novo heterozygous deletions solely affecting *FOXP1* (Horn et al. 2010). A large 1.3 Mb deletion affecting *FOXP1* and other genes was also found in a control individual (Horn et al. 2010). The three patients had moderate intellectual disability (IQ of <50), gross motor delay and a severe speech and language defect, characterised by a delay in the onset of speech, dysgrammatism and very poor speech articulation. Two of the reported patients also had an oromotor defect, including difficulties with lip protrusion and tongue elevation, but DVD was not diagnosed. These findings provided the first compelling evidence that disruptions in the *FOXP1* gene can be causative for multiple neurodevelopmental abnormalities, which include language impairment, implicating *FOXP1* in more widespread cognitive processes than were previously described for *FOXP2.* This hypothesis gained further support from a recent independent study of 110 individuals with intellectual disability and/or autism spectrum disorder (ASD), which identified a de novo intragenic *FOXP1* deletion in a patient with both intellectual disability (IQ of 58) and severe language impairment, particularly affecting expressive language (Hamdan et al. 2010). The deletion included sequences corresponding to the leucine zipper and zinc finger domains, which are important for FOXP1 dimerization and transcriptional repression. In addition to the deletion, a de novo nonsense mutation, R525X, was found in the *FOXP1* forkhead domain in another patient with non-syndromic intellectual disability (IQ of 48), severe language impairment and ASD. This study added further weight to previous findings that *FOXP1* is important for language development and normal intelligence and the discovery of a *FOXP1* stop mutation in a patient with ASD was exciting, as it highlighted *FOXP1* for the first time as a potential ASD candidate gene.

Whole exome sequencing has the potential to identify all coding variants in an affected individual and was recently used to define rare de novo coding mutations in 20 individuals with ASD and their healthy parents, along with 20 unrelated ethnically matched controls (O’Roak et al. 2011). Among several presumably causative mutations in different genes was a single base insertion in *FOXP1*, introducing a frameshift and premature stop codon (A339S*fs*X4). This individual had severe ASD and delayed language development together with intellectual disability (IQ of 34), reminiscent of previously reported *FOXP1* phenotypes (Carr et al. 2010; Hamdan et al. 2010; Horn et al. 2010). An additional inherited missense variant, H275R, was also detected in the *CNTNAP2* gene. As *CNTNAP2* has been implicated in intellectual disability (Gregor et al. 2011; Zweier et al. 2009), ASD (Alarcon et al. 2008) and in language impairment without ASD (Vernes et al. 2008; Whitehouse et al. 2012), it could also have contributed to the patient’s phenotype. As FOXP1 was found to regulate *CNTNAP2* expression, the *FOXP1* mutant identified in this patient could enhance any potentially damaging effects of the *CNTNAP2* H275R variant, highlighting the presence of putative modifier gene effects (O’Roak et al. 2011).

Very recently, direct sequencing of balanced chromosomal breakpoints in 38 patients with ASD uncovered a translocation breakpoint affecting *FOXP1* on chromosome 3p and *ANK3* on chromosome 10q in an individual with ASD and speech delay (Talkowski et al. 2012). While *ANK3* has previously been implicated in bipolar disorder and schizophrenia (Ferreira et al. 2008; Williams et al. 2011), secondary CNV analysis of *FOXP1* and *ANK3* in 19,566 patients with ASD revealed a significant CNV burden for *FOXP1*, but not *ANK3* (Talkowski et al. 2012). The distinct but complementary approaches of chromosomal breakpoint sequencing followed by an extensive secondary assessment of CNV findings provided further compelling support that *FOXP1* represents a gene with a widespread role in neurodevelopmental processes.

Taken together, five *FOXP1* single gene deletions (Carr et al. 2010; Hamdan et al. 2010; Horn et al. 2010), two intragenic nonsense and frameshift mutations (Hamdan et al. 2010; O’Roak et al. 2011) and a chromosomal breakpoint disrupting *FOXP1* supported by secondary CNV analysis (Talkowski et al. 2012) in patients with intellectual disability, ASD, language disorder and motor development delay, provide strong evidence for *FOXP1* underlying various cognitive phenotypes (summarised in Table 2).

## Similarities and differences between phenotypes associated with *FOXP1* and *FOXP2* disruptions

### Language disorder

**Fig. 1:**
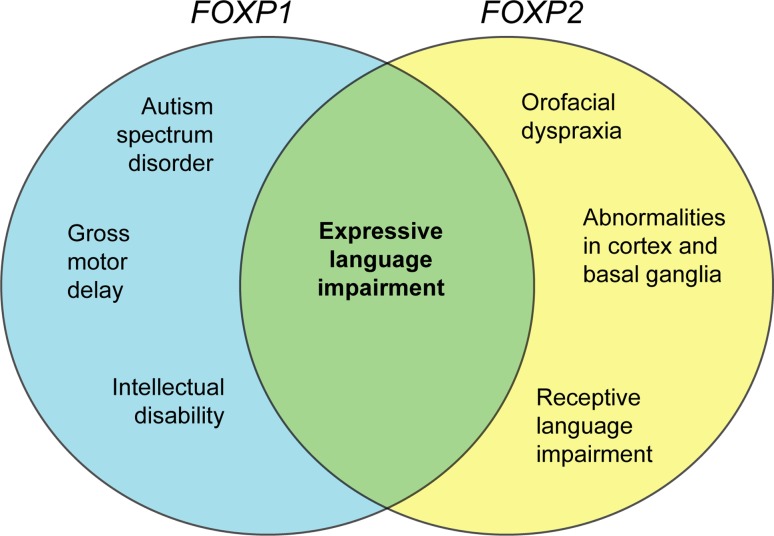
Summary of similarities and differences between *FOXP1* and *FOXP2* neuronal phenotypes

In language disorders, a distinction is often made as to whether the language impairments are primarily receptive (hearing, reading and comprehending) or expressive (speaking and writing), or affect both. People with *FOXP2* mutations have both impaired expressive and receptive language (MacDermot et al. 2005; Watkins et al. 2002a), whereas expressive language appears to be more affected in patients with *FOXP1* disruptions (Hamdan et al. 2010; Horn et al. 2010). A core feature of the phenotype associated with *FOXP2* dysfunction is an abnormal articulation due to verbal dyspraxia, i.e. impairment in the motor programming of orofacial movements required to produce normal speech (MacDermot et al. 2005; Vargha-Khadem et al. 1995). Although articulation problems have been described in some patients with *FOXP1* disruption (Horn et al. 2010), orofacial dyspraxia has not been diagnosed so far (Carr et al. 2010; Hamdan et al. 2010; Horn et al. 2010; O’Roak et al. 2011), neither have any *FOXP1* variants been identified in the small DVD patient cohort (Vernes et al. 2009). As *FOXP1* variants are yet to be associated solely with a language disorder without additional cognitive phenotypes, the possibility exists that the language disorder observed in patients with disruptions in *FOXP1* is a consequence of more global cognitive disruption rather than a specific disruption in those neural circuits necessary for speech production. This could be elucidated by comparative brain imaging of patients with *FOXP1* and *FOXP2* disruptions as well as comparing behavioural, electrophysiological and morphological phenotypes of Foxp1 and Foxp2 knockout mice. Generally, a delay in language acquisition is often observed in ASD individuals along with significantly impaired comprehension, articulation and grammar, which improves with time (Boucher 2011). Similarly, language deficits are often severe in children diagnosed with intellectual disability (Kaufman et al. 2010). Thus, the impaired language observed in patients with *FOXP1* disruptions could be a secondary consequence of these disorders. In summary, there is an overlap in the language phenotype of patients with disruptions in *FOXP1* and *FOXP2*, particularly regarding expressive language impairment (Fig. 1), but how similar the mechanistic basis is remains unclear.

### Autism spectrum disorder

Autism spectrum disorder is characterised by impaired social interaction and communication, language impairment and the presence of restrictive and repetitive behaviours. In most cases, ASD is inherited and the specific genetic disruptions are known in only a minority of cases (Geschwind 2011). ASD has been diagnosed in patients with various disruptions in the *FOXP1* gene (Hamdan et al. 2010; O’Roak et al. 2011; Talkowski et al. 2012), implicating *FOXP1* in the pathology of ASD. Chromosome 7q31 is a known autism susceptibility locus, particularly involving the language impairment of the disorder (Alarcon et al. 2002), therefore *FOXP2* has been suggested as a potential ASD candidate gene. However, *FOXP2* screenings in different cohorts of ASD patients have failed to identify any causative mutations (Gauthier et al. 2003; Newbury et al. 2002; Wassink et al. 2002). Yet, in a recent large-scale haplotype mapping analysis *FOXP2* was identified as a novel ASD candidate gene, among 1,218 others (Casey et al. 2011). *FOXP2* is also implicated in the etiological pathways of ASD through its target genes *MET* (Mukamel et al. 2011) and *CNTNAP2* (Vernes et al. 2008), although *CNTNAP2* has also been linked to language impairment where there is no diagnosis of ASD (Vernes et al. 2008). Additionally, people with isolated mutations of *FOXP2* do not show any signs of ASD even though FOXP2 is involved in pathways relevant to autism. Thus, while the possibility is open that screening studies of *FOXP2* in larger cohorts of ASD patients could identify causative *FOXP2* variants in the future, existing evidence suggests that—in contrast to *FOXP1*—*FOXP2* is not involved in ASD.

### Intellectual disability

Intellectual disability is a cognitive disorder characterised by an IQ of <70 (Kaufman et al. 2010). All non-verbal IQs reported in patients with *FOXP1* variants are below this threshold (Table 2) indicating that intellectual disability is a consistent feature of the phenotype associated with *FOXP1* disruption. Most but not all affected KE family members with *FOXP2* missense mutations have a lower IQ than unaffected members (Fisher et al. 1998, 2003; Vargha-Khadem et al. 1995; Watkins et al. 2002a) but normal IQs have been reported in other patients with perturbations in the *FOXP2* gene (MacDermot et al. 2005; Tomblin et al. 2009). Intellectual disability therefore cannot be considered a reliable characteristic of the phenotype associated with *FOXP2* disruption at this time. The reported IQs of patients with *FOXP1* disruptions are much lower than those reported for people with *FOXP2* variants (Tables 1, 2); therefore intelligence appears to be influenced more strongly by *FOXP1* than *FOXP2* dysfunction.

### Motor development delay

Although difficulties with fine motor movement and co-ordination are sometimes present in DVD patients with *FOXP2* mutations, gross motor and developmental delay appear to be more predominant in patients with *FOXP1* disruption (Tables 1, 2). The delayed motor development described in most human patients with disruptions in the *FOXP1* gene (Carr et al. 2010; Hamdan et al. 2010; Horn et al. 2010; Pariani et al. 2009) is interesting, as Foxp1 defines the identity of motor neurons in the mouse spinal cord and influences motor neuron migration, axon projection and axonal branching at muscle targets (Dasen et al. 2008; Palmesino et al. 2010; Rousso et al. 2008). Therefore the delayed motor development in individuals with *FOXP1* disruptions may be influenced by pathologies in both the central and peripheral nervous system.

## Steps towards elucidating neurodevelopmental pathways influenced by *FOXP1* and *FOXP2*

Disruptions of *FOXP1* and *FOXP2* cause distinct phenotypes with some overlapping features (Fig. 1), pointing to both shared and distinct neurodevelopmental roles for these two genes. Below we discuss potential approaches to elucidate the molecular pathways and circuits involved.

### Defining the neural phenotype of patients

The neural basis of behavioural abnormalities can be identified by structural and functional brain imaging of patients. MRI analyses of some *FOXP1* patients have been performed but have revealed no obvious structural brain abnormalities (Carr et al. 2010; Horn et al. 2010). Conventional neuroradiological assessment of MRI scans performed on affected members of the KE family also revealed no obvious abnormalities at first (Vargha-Khadem et al. 2005), but application of voxel-based morphometry, which can detect more subtle differences in grey and white matter and additional volumetric analysis revealed significant differences in specific brain regions of affected KE members (Watkins et al. 2002b). The neuroanatomical effects of the KE mutation have been extensively reviewed elsewhere (Vargha-Khadem et al. 2005), but in brief involve abnormalities in the cortex, cerebellum and basal ganglia, particularly the striatum (Liegeois et al. 2003; Vargha-Khadem et al. 1998; Watkins et al. 2002b). These findings from brain imaging implicate *FOXP2* in the development of cortico-striatal circuits, which are involved in sensorimotor integration required for vocal motor learning. This would suggest that the phenotype observed in the KE family stems from disruption in the underlying neural circuits of language development, an idea that has received support from animal studies (see below).

### Animal studies

Studies using animal models have taken us a long way towards elucidating the role of Foxp2 in brain development. Several Foxp2 knockout and mutant mouse models have been generated and the different phenotypes have already been extensively reviewed (Fisher and Scharff 2009). In brief, these phenotypes include developmental delay, motor impairment, cerebellar abnormalities and disrupted synaptic plasticity in the striatum. Recently, it was further demonstrated that motor skill learning is significantly impaired in mice carrying the equivalent mutation to that found in affected KE family members, which was explained by aberrant striatal activity during motor skill learning (French et al. 2011).

**Fig. 2:**
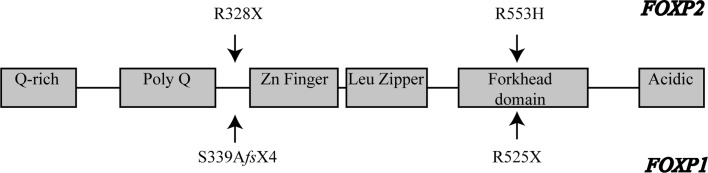
Summary of different *FOXP1* (*below*) and *FOXP2* (*above*) mutations decribed. See Tables 1 and 2 for reference details. Variant S339AfsX4 was identified by whole exome sequencing and an additional missense variant in the *CNTNAP2* gene was also present in this individual

Two amino acid substitutions in the human FOXP2 protein have emerged during human evolution, possibly linked to the development of speech and language (Enard et al. 2002) and a partially humanised form of the *FOXP2* gene has been shown to increase synaptic plasticity in the striatum of mice (Enard et al. 2009). This phenotype is opposite to what has been described in mice with Foxp2 dysfunction (French et al. 2011; Groszer et al. 2008) suggesting that the emergence of human FOXP2 may have enhanced cortico-striatal circuits or circuit properties during the evolution of spoken language. Taken together, these animal studies have revealed that Foxp2 is involved in the development of neural plasticity in circuits contributing to language and a possibly wider cognitive function, which is in agreement with the findings from neuroimaging of KE family members (see above) (Fig. 2).

Foxp1 knockout mouse models have been used to investigate the importance of Foxp1 in a range of non-neural developmental processes (Feng et al. 2010; Hu et al. 2006; Shu et al. 2007; Wang et al. 2004; Wu et al. 2006) and in the development of motor neurons in the spinal cord (Dasen et al. 2008), but have not been used to define the role of Foxp1 in brain development. Homozygous loss of Foxp1 causes embryonic death at E14.5 due to heart failure (Wang et al. 2004). Conditional removal of Foxp1 in the brain will therefore be the key to elucidating the importance of Foxp1 during later stages of brain development. It will be interesting to see whether brain-related aspects of the Foxp1 knockout phenotype are distinct from the Foxp2 knockout phenotype, as is the case in the human situation. *Foxp1* and *Foxp2* expression overlaps in the striatum, thalamus, superior colliculus and inferior olive in the mature mouse brain, but their expression differs in other regions including the cortex, hippocampus and inferior colliculus (Ferland et al. 2003). It is interesting that histological analyses of brains from Foxp2 knockout mice only revealed gross morphological abnormalities in the cerebellum, where Foxp1 is not co-expressed (French et al. 2007), suggesting that Foxp1 may compensate for the loss of Foxp2 and that a level of redundancy exists between these two genes. It will be interesting to see whether layers 3, 4 and 5 of the cortex or the hippocampus which express Foxp1 only are exclusively abnormal in the Foxp1 knockout mouse.

### Identification of target genes

Common FOXP1 and FOXP2 neurodevelopmental pathways seem likely, considering their potential to form heterodimers and their co-expression in certain brain tissues. On the other hand, the distinct phenotypes seen in patients with *FOXP1* and *FOXP2* disruption would suggest they participate at least to some degree, in independent pathways. The identification and classification of target genes hold the key to determining which pathways and networks involve FOXP1 and FOXP2 during brain development. For FOXP2, genome wide in vivo ChIP-chip screens coupled to expression profiling have already been used to define transcriptional targets in the developing brain; many of these targets were found to be involved in pathways regulating neurite outgrowth, axon guidance and synaptic plasticity (Konopka et al. 2009; Spiteri et al. 2007; Vernes et al. 2007, 2011). Elsewhere, it has been shown that FOXP2 directly regulates *CNTNAP2*, mutations in which, like *FOXP2*, were found to be linked to language impairment (Vernes et al. 2008), the autism candidate gene *MET*, involved in neuronal differentiation (Mukamel et al. 2011) as well as the schizophrenia susceptibility gene *Disrupted in schizophrenia 1* (DISC1) in HEK293 cells (76). Whether the identification of DISC1 as a FOXP2 target places FOXP2 in schizophrenia-related pathways remains unclear. There is conflicting evidence for a role of *FOXP2* in schizophrenia; significant associations have been reported between certain *FOXP2* SNPs and schizophrenia (Sanjuan et al. 2006; Tolosa et al. 2010), but the *FOXP2* missense and nonsense mutations associated with DVD (Table 1) were not found in patients with schizophrenia (Sanjuan et al. 2005). In conclusion, the published targets of FOXP2 implicate this transcription factor in pathways regulating neurite outgrowth, axon guidance and synaptic plasticity, which is in agreement with a role of FOXP2 in the development of neural circuits for language development.

The genes regulated by FOXP1 in developing brain tissue have not yet been identified by genome-wide ChIP screens. Overexpression of Foxp1 in a murine striatal cell line has recently revealed a repression of immune-related genes (Tang et al. 2012), which is in agreement with previous studies demonstrating a role of Foxp1 in immune development. Given the level of cognitive disruption caused by *FOXP1* variants, it is reasonable to assume that FOXP1 targets will be uncovered in many neurodevelopmental pathways and not limited to immune function. There is evidence that *CNTNAP2* expression is regulated by FOXP1 (O’Roak et al. 2011), which has also been shown for FOXP2 (Vernes et al. 2008), providing the first evidence for a common FOXP1–FOXP2 pathway involving CNTNAP2 in neurodevelopmental processes. On the other hand, the fact that *CNTNAP2* is implicated in both ASD (Alarcon et al. 2008) and in language impairment without ASD (Vernes et al. 2008) suggests distinct CNTNAP2 pathways exist. Elsewhere, Foxp1 has also been shown to regulate *Pitx3* transcription during the differentiation of ES cells into midbrain dopaminergic neurons (Konstantoulas et al. 2010).

Typically, transcription factors do not act alone but rather in complexes together with other transcription factors, chromatin remodellers and cofactor proteins to bind to promoter sequences and regulate target gene expression. FOXP1 and FOXP2 interact with various proteins to regulate gene expression in different tissues (supplementary Table 1) (Chokas et al. 2010; Datta et al. 2008; Jepsen et al. 2008; Li et al. 2004; Otaegi et al. 2011; Ravasi et al. 2010; Takayama et al. 2008; Wu et al. 2006; Zhou et al. 2008) but identification of the combinatorial interactions of FOXP1 and FOXP2 with other proteins specifically in the brain will be needed to understand the roles they play in brain development. The interactions of FOXP1 and FOXP2 with different proteins represent another dimension to the regulation of their target genes.

## Concluding remarks

Disease-causing variants in both *FOXP1* and *FOXP2* are relatively rare, but play a significant role in the pathology of cognitive diseases. While the importance of *FOXP2* in the pathology of DVD has been a popular topic of investigation for more than 10 years, it remains to be seen whether perturbations in *FOXP2* are confined to language impairment or whether there is also some risk for other diagnostic entities. Genetic evidence emerging in the last 2 years provides compelling evidence that *FOXP1* variants are responsible for a more global cognitive phenotype, encompassing language impairment, intellectual disability, ASD and motor development delay. The phenotypic spectra of *FOXP1* and *FOXP2* disruptions suggest that these two closely related transcription factors are involved in both shared and distinct neurodevelopmental pathways underlying cognitive diseases through the regulation of common and exclusive targets. The findings discussed in this review show that *FOXP1* and *FOXP2* may provide crucial insight into the molecular pathways involved in human cognitive diseases.

## Electronic supplementary material

Below is the link to the electronic supplementary material.

Supplementary material 1 (DOC 36 kb)
