# Supplementary material for: The distinct and overlapping phenotypic spectra of FOXP1 and FOXP2 in cognitive disorders
Source: Hum Genet. 2012 Jun 27;131(11):1687–98. doi: 10.1007/s00439-012-1193-z (PMC3470686; doi:10.1007/s00439-012-1193-z)
Supplement: Supplementary file 1 — Supplementary material 1 (DOC 36 kb) [file 439_2012_1193_MOESM1_ESM.doc]

| **FOXP1** | | | **FOXP2** | | |
| --- | --- | --- | --- | --- | --- |
| **Reference** | **Interaction Partner(s)** | **Tissue** | **Reference** | **Interaction Partner(s)** | **Tissue** |
| Li et al. 2004 | FOXP1, 2, 4 *(TF)*  CtBP1 *(CR)* | Lung | Li et al. 2004 | FOXP1, 2, 4 *(TF)*  CtBP1 *(CR)* | Lung |
| Takayama et al. 2008 | Androgen receptor *(TF)* | Prostate |
| Jepsen et al. 2008 | SMRT *(CR)* | Heart | Wu et al. 2006 | NFAT *(TF)* | T cells |
| Datta et al. 2008 | micro RNA-1 | Liver |
| Chokas et al. 2010 | P66ß and HDAC2 *(CRC)* | Lung | Zhou et al. 2008 | Nkx2.1 *(TF)* | Lung |
| Ravasi et al. 2010 | Tle6, Tcfe2a, Phf2, Per2, Per1 and Smad4 | CHO K1 cells |
| Otaegi et al. 2011 | micro RNA-9 | Spinal motor neurons |

**Supplementary Table 1** Summary of protein interaction partners of FOXP1 and FOXP2. TF, - transcription factor; CR - co-repressor; CRC - components of chromatin remodelling complex.
